# Epigenetics of Trauma Transmission and Fetal Alcohol Spectrum Disorder: What Does the Evidence Support?

**DOI:** 10.3390/ijerph20176706

**Published:** 2023-09-03

**Authors:** Sarah M. Orton, Kimberly Millis, Peter Choate

**Affiliations:** 1 Faculty of Science and Technology, Department of Biology, Mount Royal University, Calgary, AB T3E 6K6, Canada; kmill222@mtroyal.ca; 2 Faculty of Health, Community & Education, Department of Child Studies and Social Work, Mount Royal University, Calgary, AB T3E 6K6, Canada; pchoate@mtroyal.ca

**Keywords:** fetal alcohol spectrum disorder, FASD, prenatal alcohol exposure, PAE, epigenetics, trauma, epigenetic inheritance, intergenerational trauma, transgenerational trauma, social work

## Abstract

Fetal alcohol spectrum disorder (FASD) results from teratogenic impacts of alcohol consumption during pregnancy. Trauma and prenatal alcohol exposure (PAE) can both cause neurodevelopmental impairment, and it has been proposed that FASD can amplify effects of trauma. Certain PAE and trauma effects are mediated via epigenetic mechanisms. The objective of this review is to present the current evidence for epigenetics in trauma transmission as it relates to FASD, to help bridge a potential knowledge gap for social workers and related health professionals. We include a primer on epigenetic mechanisms and inheritance, followed by a summary of the current biomedical evidence supporting intergenerational and transgenerational epigenetic transmission of trauma, its relevance to FASD, the intersection with social transmission, and finally the application to social work. We propose potential models of transmission, considering where social and epigenetic pathways may intersect and/or compound across generations. Overall, we aim to provide a better understanding of epigenetic-trauma transmission for its application to health professions, in particular which beliefs are (and are not) evidence-based. We discuss the lack of research and challenges of studying epigenetic transmission in humans and identify the need for public health interventions and best practices that are based on the current evidence.

## 1. Introduction

Alcohol has a long-standing place in society but is now recognized as a teratogen in pregnancy that can be linked to lifelong impairments for a child exposed in utero. Alcohol use in pregnancy is a global problem. Despite multiple efforts to promote abstinence in pregnancy, a significant number of pregnant women continue to consume alcohol. A population survey in the USA found that 10.2% of the surveyed population had consumed any alcohol during pregnancy, with approximately 3% reporting binge drinking [1]. More globally, based on findings from a meta-analysis, it is estimated that 9.8% of pregnancies worldwide are alcohol-exposed [2].

Fetal alcohol spectrum disorder (FASD) results from the teratogenic impact of alcohol consumption during pregnancy. It is the leading preventable cause of congenital physical and cognitive disabilities in Canadians [3]. Children with FASD represent approximately 3–11% of all children involved with child protection in Canada [4]. Individuals with FASD are affected by traumatic childhood experiences (trauma), such as abuse and neglect, at disproportionately high rates [5]. Trauma and prenatal alcohol exposure can both cause neurodevelopmental impairment, but the combined effects have not been well studied [6]. It has been hypothesized that children born with FASD are more susceptible to the impact of trauma, causing greater than expected cognitive and developmental deficits compared to either factor alone [6]. One plausible mechanism for this is that prenatal alcohol exposure alters the epigenome to invoke an increased stress response, making these individuals more susceptible to trauma effects [7]. What is not clear is whether there are also inherited trauma-related epigenetic modifications that are *not* in utero epigenetic effects and that are distinct from the effects of alcohol—if present, they could potentially be transmitted to future generations, although there is no known mechanism for this to date.

The trauma experienced by children diagnosed with FASD is complex and reflects intergenerational transmission (F0 to F1), with similarities in central nervous system impairments observed in children with histories of prenatal exposure or trauma [8,9,10]. It can be difficult to tease out the origin of trauma-related impacts. Often, trauma experiences in FASD are attributed to parental trauma, which impacts parenting capacity [6]. In addition, early-life maltreatment trauma is believed to be a risk factor for maltreatment of future offspring [11]. These types of behavioural or social transmission of trauma are difficult to separate from effects of epigenetic transmission, which we will discuss in more detail. Understanding the framework of FASD–trauma–epigenetics would help inform interventions and policy in social work. 

Epigenetic modifications are alterations in deoxyribose nucleic acid (DNA) that do not change the DNA sequence but regulate which genes are expressed. They can provide both short-term and long-term changes to a cell’s activity. The most studied epigenetic mechanism is DNA methylation, which involves the addition of a methyl group to cytosine residues, and they are particularly important at promoter regions, which control whether genes are active or not [12]. Epigenetic signatures are considered stable, such as marks that cause cell specialization, but are also flexible enough to adapt to changing environments. 

The question of whether aspects of trauma and FASD have epigenetic markers that can be transmitted across generations has been raised in the literature [13,14,15]. This idea of transgenerational trauma transmission via epigenetic mechanisms has been picked up in popular literature and media [16]. While the media may note some of the related scientific controversy, many people often accept the media claims at face value, leading to beliefs that transgenerational inheritance or “ancestral trauma memory” has been validated by scientists. Other writers correctly assert that it is difficult to tease out transgenerational social transmission as opposed to biological transmission, or some combination of both [17]. 

Interventions to prevent FASD, as well as to minimize its frequency and support mothers at risk and those impacted by the disorder, are important to reduce both prevalence and impact. In certain countries, social work is often called upon to work with this population in the diagnostic, support, and prevention environments. An understanding of how trauma transmission may or may not be related to epigenetics is important in social work, in order to better understand how interventions can be structured. 

The objective of this review is to present the supporting scientific evidence for epigenetic influences in trauma transmission, as it relates to FASD, and explain the current limitations and lack of research investigation in this field. The content explanation is targeted towards those in social work and related health professions, to help bridge the gap using evidence from basic and clinical sciences literature. Because of the interdisciplinary nature of the content and target audience, this paper provides background information in epigenetics, epigenetic mechanisms, as well as epigenetics in FASD and trauma. We then discuss social transmission of trauma, how this intersects with biological transmission, and the relevance to social work and other health professions. Potential models of transmission, considering where social and epigenetic pathways may intersect and/or compound across generations are proposed. We discuss which epigenetic-trauma transmission beliefs are not yet evidence-based and what research is still limited and identify pathways to improve communication to front-line practitioners and others who support people with FASD.

## 2. Methods

A traditional literature review was deemed the most appropriate approach given the broad scope and multi-disciplinary nature of the content covered. More specifically, we reviewed literature in the sciences and social sciences, then its application within social work and related fields. A key aim was to provide an overview of the current state of the published literature, including discerning whether current perspectives and views are supported by scientific evidence in the fields of cellular and molecular biology. Databases (APA PsychInfo, MEDLINE, PubMed, Google Scholar, Academic Search Complete) were searched for relevant articles that connected at least two of the following key terms: “Fetal Alcohol Syndrome Disorder”, “trauma”, “epigenetics”, “intergenerational trauma”, and “transgenerational trauma”. It is important to note that there were no articles found in PubMed/Medline when using the three index terms “Fetal Alcohol Spectrum Disorder”, “trauma”, and “epigenetics”. Inclusion criteria were papers published between 1970 and 2023 and written in English. For scientific studies, we aimed to focus on human studies; however, due to the scarcity of human subject clinical papers, it was important to include benchmark animal studies to evaluate epigenetic transmission content. For the scientific publications, only peer-reviewed articles were included. For the social sciences content, journals, reports, policy and government documents, and theses were included in the search. Figures were created using BioRender (Biorender.com), Lucidchart (Lucidchart.com), and Powerpoint.

## 3. FASD and Prevalence 

Alcohol is a neurotoxic teratogen that travels through the mother’s bloodstream and readily crosses the placenta. Alcohol is eliminated slower from the placenta than other tissues and accumulates in the amniotic fluid where prolonged exposure interferes with the development of the embryo [18]. 

Fetal alcohol spectrum disorder (FASD) is the name given to the broad spectrum of physical, neurological, endocrinological, behavioural cognitive, and neurodevelopmental deficits resulting from alcohol exposure during gestation [19]. The most severe form of FASD is fetal alcohol syndrome (FAS) at one end of the spectrum, with milder potentially undiagnosed symptoms at the other end. The resulting deficits in FASD depend on the quantity of alcohol exposure, duration, and the timing during gestation, but also on genetic factors, maternal nutrition, and maternal health [20]. 

Relatively speaking, FASD is not a diagnosis with a long history. In 1968, the French physician Paul Lemoine described facial dysmorphic features and developmental concerns in children whose mothers had consumed wine in pregnancy [21]. This was followed by the work of David Smith and Ken Jones who also identified facial features and physical developmental concerns in children whose mothers had consumed alcohol in pregnancy [22]. This research group identified patterns in FASD expression, although it focused mainly on significant cases [22]. Recent work by Himmelreich, Lutke, and Hargrove [23] has shown that those with FASD may experience a number of early-onset physical disorders at a rate much higher than would be seen in the general population. The combined view of the research indicates those affected with FASD commonly experience disruption in any to all of the following: cognitive function, learning and memory, attention deficit–hyperactivity disorder (ADHD), communication, impulse control, self-regulation, mood, sleep regulation, response to stress, and social interactions [22,24,25,26,27,28,29,30]. While the underlying molecular mechanisms manifesting these symptoms are poorly understood, many of them are mediated (at least to some extent) by epigenetic alterations resulting from prenatal alcohol exposure.

A report published by the Centre for Addiction and Mental HealIh (CAMH) in 2019 found the prevalence of FASD in elementary-aged students in Toronto, Canada, was 2–3% [3]. Popova and colleagues [3] suggest that the frequency might be much greater when the general population is considered, which would include children showing less severe symptoms and less likely to be formally diagnosed. These findings were supported by US data which found a conservative estimate of 1.1% to 5.0% when surveying first-grade students in four communities [31]. It is believed that the reported prevalence numbers for FASD may be higher than diagnostic figures due to misdiagnosis, alternative diagnostic features such as learning disabilities, and behavioural concerns, as well as concerns with executive function and sensory, motor, visual, and spatial memory [21,32,33]. Studies have indeed reported the presence of prenatal alcohol exposure (PAE) occurring in both FASD and autism spectrum disorder (ASD), along with overlapping diagnostic features [34,35,36,37,38]. Some diagnostic confusion may be in part driven by the greater social acceptance of ASD as opposed to FASD [38,39].

Lange and colleagues conducted a systematic and meta-analysis of FASD worldwide [40], including 24 studies involving 1416 children and youth. They reported an estimated FASD prevalence of 7.7/1000 globally, with significant worldwide variation [40]. The World Health Organization (WHO) European region had the highest regional rates at 19.8/1000 and the WHO Eastern Mediterranean Region the lowest at 0.1/1000 [40]. There were notable prevalence figures in specific countries, such as South Africa (111.1/1000), Croatia (53.3/1000), and Ireland (47.5/1000). Their study also analyzed population subsets, observing much higher prevalence rates in incarcerated, adopted and foster children, orphans, and psychiatric populations, as well as Indigenous populations. This raises the question of the linkages to trauma [6,8,9,10,11,41].

## 4. What Is Epigenetics?

Epigenetics is often defined as heritable changes in gene expression, without changes to the sequence of the DNA [42]. Transmission of epigenetic modifications to daughter cells (mitosis) or to offspring (meiosis) is considered epigenetic inheritance. It involves changes to gene expression as a result of DNA methylation, histone modifications (acetylation, methylation, phosphorylation, sumoylation, and more), and non-coding RNAs. 

The term epigenetics was first coined by Charles Waddington, who suggested the possibility of an organism expressing different traits based on the environmental stimulus to which it is exposed [43]. It came from merging the terms “epigenesis” and “genetics”, roughly translating to “upon or above the genome”—in other words, making a change “on” the genome without altering the sequence itself [44]. Since then, the modern definition has redefined epigenetics as the changes in gene expression, or phenotype, that are not caused by changes in the DNA sequence [42,45]. 

Epigenetics is the study of how the environment affects expression of a phenotype, in a heritable manner, without changing the underlying genotype [46,47]. The collection of epigenetic tags, or marks, is referred to as the “epigenome” [46]. Numerous epigenetic studies have been conducted on various animal species in the last two decades, and more recently human studies, to examine the mechanisms by which the environment can produce or alter the epigenome. Substance abuse, stress, and maternal care are just some of the environmental factors studied with respect to their impact on epigenetic markers. 

## 5. Epigenetic Mechanisms

Section 5 and Section 6 are aimed at readers who would like to learn more about the science of epigenetic effects. 

The main mechanisms of epigenetic modifications that have been identified include DNA methylation, post-translational histone modifications, and RNA-associated gene silencing by non-coding RNAs [48,49]. A detailed molecular explanation of how these processes work is beyond the scope of this paper; however, a condensed and simplified primer on these three main epigenetic mechanisms (DNA methylation, histone modification, and non-coding RNA) is given here. 

**Figure 1:**
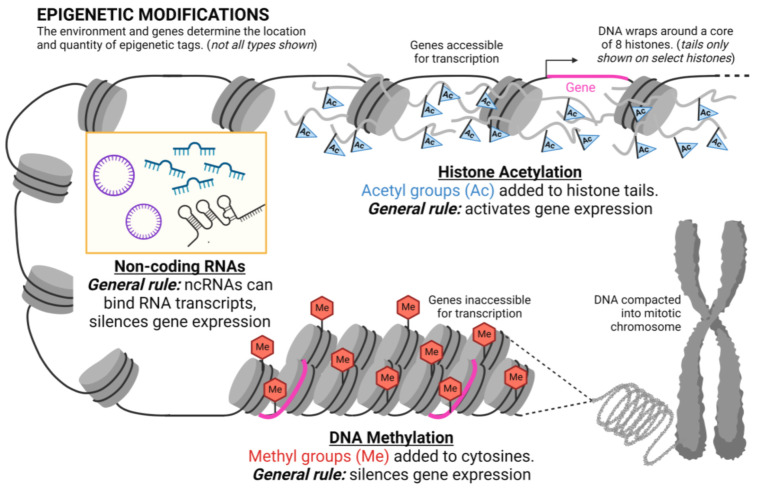
Main types of epigenetic modifications inside the nucleus of a cell, illustrating how gene expression is regulated via DNA methylation, histone acetylation, and non-coding RNAs. The sum of all epigenetic markers makes up the epigenome, which varies by cell type and between individuals. The epigenome can be modified by certain environmental exposures. Me—methyl; Ac—acetyl; nc—non-coding.

Higher-level multicellular organisms, such as humans, are composed of trillions of cells, cells being the fundamental units of life. The nucleus within a cell houses our DNA, which contains the instructions for the structure and functioning of a cell. The DNA is packaged as chromatin, where it wraps around proteins, called histones, to help condense and organize the DNA within the nucleus. The first step of chromatin formation involves the DNA wrapping around a core of eight histones to create a nucleosome (Figure 1). These bead-like structures will coil and condense even further to allow for very efficient packaging of DNA [50]. 

Both the histones and the DNA itself will undergo epigenetic modifications that will alter this packaging (chromatin structure) to regulate gene expression. DNA methylation is the addition of a methyl group (CH_3_) to certain carbons of cytosine residues in DNA (Figure 1). It is the most researched and prominent epigenetic mechanism. Methylated cytosine residues occur next to guanine residues, such that methylation will be mirrored on both sides of the DNA double helix. DNA methyltransferases (DNMT) are the enzymes responsible for this methylation (adding methyl groups). Cytosine residues throughout the genome can be methylated, but the most important type of methylation occurs within promoter regions of genes. Genes are sections of DNA that will be transcribed into RNA, and coding RNAs are then translated into proteins. Transcription of a gene is initiated at the promoter region, many of which contain CpG islands—prominent stretches of CpG (cytosine-phosphate group-guanine) [51]. When CpG islands in promoters are methylated, it allows for the recruitment of gene expression repressors. As such, DNA methylation is generally associated with silencing genes (Figure 1).

Histone proteins can also be epigenetically modified. Post-transcriptional modification to histones involves adding acetyl, methyl, SUMO (small ubiquitin-like modifier), and/or phosphate groups to a part of the proteins, called the tails. These modifications to histones act as epigenetic tags that help to remodel chromatin and regulate the expression of genes in the vicinity of the tags. There are many epigenetic marks added to the histones, and it is the sum of these marks in the epigenome that helps determine whether a particular gene will be expressed or repressed. For example, high histone acetylation is associated with a form of accessible chromatin (euchromatin) that promotes expression of genes within that area (Figure 1). As such, histone acetylation generally has the opposite effect on high DNA methylation (high histone acetylation allows for increased gene expression; high DNA methylation allows for decreased gene expression), although there are various exceptions to this general rule. 

More recently, long-lived non-coding RNA (ncRNA) transcripts have been identified as an epigenetic mechanism in gene regulation and potentially in the transmission of the epigenome in offspring [52,53,54] (Figure 1). These include microRNAs (miRNAs), long-non-coding RNAs (lncRNAs), and transfer-RNA-derived small RNAs (tsRNAs), among others. Some non-coding RNAs can sequester mRNA transcripts, preventing their expression, while others are able to recruit enzymes involved in regulating expression. This is a rapidly emerging field in molecular biology, and many of the epigenetic effects of ncRNAs on the regulation of gene expression are still being elucidated. 

Of these different epigenetic mechanisms, DNA methylation is recognized as the most stable epigenetic modification and therefore is the most studied [55] when looking at potential transmission across generations.

## 6. What Is Epigenetic Inheritance?

Epigenetic inheritance is classified as the stable transmission of epigenetic modifications influencing a phenotypic trait to offspring; it relates to cells dividing within an individual (mitosis) and producing offspring (meiosis) [42,45,49]. 

All our cells, with a few exceptions, contain the exact same DNA (the same genome). What makes them different is which genes are transcribed and translated into proteins (different proteomes)—accounting for differences in structure and function in the different types of cells within an organism. Because each type of specialized cell will have a different pattern of gene expression (accounting for its different proteome), differentiated cells need a way to remember this pattern of expression, or their identity, when they divide to form new cells. This is called cell memory.

Epigenetic modifications during development are stable and contribute to cell memory in differentiated (specialized) cells. When these specialized cells divide through mitosis, to create all of the cells in our body, they need a process of “remembering” which genes must be silenced vs. activated to contribute to their identity. For example, when a fibroblast divides, the newly synthesized daughter cells need a way to “remember” that they are also fibroblasts—this is achieved through epigenetic inheritance; identity tags like DNA methylation, histone modifications, and ncRNAs are passed on to the newly formed cells so that they are also fibroblasts. In contrast to these stable tags, many other epigenetic marks are flexible, such that they can be altered as cells adapt to the changing environmental conditions.

Epigenetics in creating a new generation does not follow the same inheritance pattern that we see in dividing cells within an individual (i.e., cell memory). During early embryonic development, the parental epigenome is almost completely erased. In humans, this “clearing away” or erasure of the parental epigenetic signature occurs during very early embryonic development, soon after fertilization has occurred [49,56]. By wiping away parental epigenetic marks, each new generation essentially starts life with a “blank slate”. This epigenome erasure is necessary to restore the developmental potency of the developing embryo [56]. In other words, by erasing the epigenetic tags from the egg and the sperm, the zygote is a stem cell that can divide and produce all the different specialized cells in our body [57]. This erasure of the parental epigenome is important when hypothesizing about transmitting exposures (i.e., trauma) across generations (discussed later). 

One exception to the “clean slate” are imprinted genes. Maternally or paternally imprinted genes somehow escape the process of epigenetic clearance, and the imprinted marks are passed down to the progeny [48,56]. These imprinted genes account for less than 1% of genes, and it is not clear whether specific imprinted marks are permitted to be transmitted, or if they are instead missed in the clearance reprogramming phases. It is important to understand that imprinting does *not* constitute a mechanism to explain transgenerational transmission. 

Following the erasure of the parental epigenome during embryonic development (apart from imprinted genes), “reprogramming” of cells occurs, whereby cells regain epigenetic modifications. This occurs after the implantation of the embryo and will determine the gene expression for each cell [48,56]. These new epigenetic markers are important in determining the fate of specialized cells, such as differentiating into an epithelial versus muscle cell. This type of stable epigenetic inheritance is required for cell memory, as an individual grows from an embryo to a fetus and all the way into adulthood. However, there are also more plastic epigenetic changes that are caused by an organism responding to its changing environment. It is these types of epigenetic markers that we discuss within this review.

Animal studies have investigated the heritability of environmentally induced epigenetic marks from one generation to the next, with varying results depending on the environmental cause of the epigenetic marker and the species studied [58,59]. Over the past two decades, the interdisciplinary field of epigenetics has gained popularity in the mainstream media, due in large part to a ground-breaking study released in 2001 [60] and another article published in Nature, 2004 [61]. The latter study was conducted by a team of Canadian scientists who studied the effects of maternal care on rat pups [61]. The study found that the pups had different stress responses, which carried into adulthood, depending on whether they had more attentive or negligent maternal care during a critical period of development (in the first 2 weeks of life) [61]. These changes were attributed to epigenetics; in other words, gene activation or inactivation triggered by environmental factors that do not change the underlying DNA sequence. The Weaver et al. [61] article became a benchmark study and has been referenced in over 4000 publications to date. It also has been erroneously represented in the media as “scientific proof” that stressful environments can cause changes in our epigenome that are inherited over several generations [62], a point discussed later in this review.

The well-known Agouti mouse model has been used to examine how environmental exposures and maternal diet can impact phenotype via epigenetic changes [63]. The Agouti gene impacts the colour of the offspring’s coat of fur. Hypomethylation of the Agouti gene, which can be caused by BPA exposure, produces yellow fur and an increased predisposition to obesity. Findings showed that a diet rich in methyl donors (i.e., folic acid or S-adenosyl methionine) could rescue this effect by increasing DNA methylation of the Agouti gene; these epigenetic changes were inherited up to two generations [58,64]. The advantage of studying epigenetic transmission in rodent models is that the laboratory environment can be controlled to a large extent, limiting the opportunity for the same environmental stressors to act de novo in each generation. Of course, the general aim of animal studies is to extrapolate findings to deduce what may be occurring in humans.

## 7. Defining Intergenerational and Transgenerational Epigenetic Transmission 

Epigenetic inheritance refers to epigenetic modifications that are transmitted either from a parent cell to daughter cells (mitosis) or to the next generation from parents to offspring (via the germline) [42,45,49]. Transgenerational epigenetic inheritance is the term used to describe an epigenetic mark that is inherited from grandparents and beyond. 

**Figure 2:**
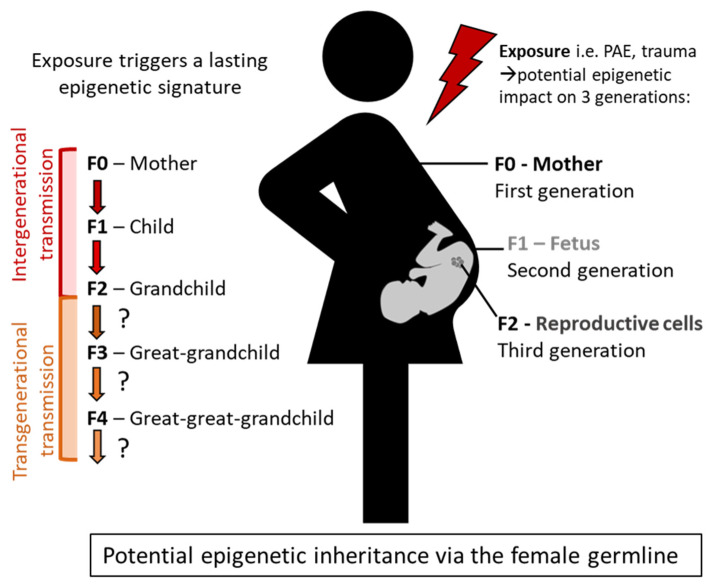
Certain environmental exposures have the potential to alter the epigenome (epigenetic modifications, such as DNA methylation and histone acetylation). The difference between intergenerational and transgenerational epigenetic inheritance caused by an environmental exposure is illustrated. Evidence for true transgenerational transmission of epigenetic tags that can survive the reprogramming phase in humans is lacking (represented by question marks). F—filial.

It is important to clarify the difference between intergenerational and transgenerational epigenetic transmission. When the offspring’s (F1) epigenome is altered due to direct exposure to an environmental factor, this is termed intergenerational epigenetics [53,54]. For example, the in utero effects on the fetus due to maternal (F0) alcohol consumption during pregnancy would be intergenerational transmission. In addition, since the alcohol exposure also can impact the eggs within the ovaries of the developing fetus, any impact seen in the second generation (F2, i.e., grandchildren) would still constitute intergenerational effects in the case of prenatal alcohol exposure (Figure 2). In order to be considered transgenerational epigenetic effects, the altered phenotype must result from indirect or preconception effects [54,65]. This would mean that an environmental stimulus creating an epigenetic mark is passed on to the second generation (F2) for male transmission or third generation for female transmission (F3, i.e., great-grandchildren) (Figure 2). While the scientific evidence supporting trauma-mediated intergenerational epigenetic effects is fairly well established, there remains a lack of evidence implicating transgenerational effects. 

If a mechanism for epigenetic transgenerational inheritance indeed existed, it would imply that acquired traits (non-genetic) could provide some type of cellular memory adapting an individual to a particular environmental condition [54]. Given the current understanding of epigenetic clearance and reprogramming mechanisms in human embryonic development, this remains a controversial hypothesis [53,66]. One of the challenges in “proving” that a phenotypic trait is truly due to transgenerational epigenome modifications in humans would be the inability to discern whether epigenetic marks were inherited versus the same marks developed de novo in the offspring.

The word transmission does not automatically imply that it is due to a biological mechanism or something biologically inherited, such as via epigenetic mechanisms. When considering transmission of psychological or physiological trauma effects in FASD, it is important to also carefully consider the influences of social and cultural transmission [67] (discussed in Section 10). 

In reviewing the literature, we encountered inconsistency within the terminology of transmission. There were studies with experimental findings supporting what is classically defined as intergenerational trauma (i.e., F0 to F1 paternal effects or F0 to F2 maternal effects) where the authors termed this as transgenerational trauma transmission, even in the title of the manuscript. Given that these terms can be unintentionally misrepresented in published molecular biology studies, it makes it even more difficult for social workers to navigate the evidence base. It may also be that there are difference by region, or related to language translations. Along the same lines, this provides popular media a platform to jump to conclusions when a study unintentionally makes claims about the transgenerational inheritance of trauma, when in fact the findings support intergenerational transmission due to a direct effect of an environmental stimulus. For example, if a trauma or addiction occurs in the biological parent and consequences are observed in the offspring (F1), this is considered intergenerational trauma due to the potential for direct exposure (Figure 2) but is often mis-named. 

The first known studies of transgenerational epigenetic inheritance were in corn in the 1950s [68,69]; however, evidence of similar mechanisms in mammals has been much more challenging to ascertain. We discuss the current evidence supporting both intergenerational and transgenerational epigenetic transmission in the following sections and relate this to trauma, FASD, and the intersection with social transmission.

## 8. Epigenetic Effects and Transmission in FASD 

Adverse in utero environmental exposures have the potential to permanently program physiological and behavioural systems, which can then have various health implications into adulthood [70,71]. One such adverse exposure is to alcohol. The molecular mechanisms underpinning the teratogenic effects of alcohol exposure are complex and poorly understood; however, evidence continues to help elucidate the role of epigenetic mechanisms [19]. It is generally accepted that prenatal alcohol exposure in utero can cause epigenetic modifications in the developing fetus [72,73,74]. These epigenetic changes in FASD occur during critical embryonic/fetal developmental stages and have varying structural, neurological, endocrinological, cognitive, and behavioural effects [70,75]. In addition, these lasting epigenetic signatures can alter an individual’s response to stress and trauma later in life [28]. The need for more FASD epigenetics research is especially salient given that the detrimental effects of prenatal alcohol exposure extend throughout the individual’s lifetime.

As a reminder, epigenetic modifications can change the way our cells behave by activating or silencing gene expression (which is how proteins are made), but they do not alter the genetic sequence itself. Studies in animal models, cultured neurons, and some early human clinical studies support the negative impact of alcohol on epigenetic programming in developing neurobiological systems (reviewed in [70]). 

Initial epigenetic studies assessed the impact of embryonic/fetal alcohol exposure on persistent changes to the transcriptome (RNA transcripts), which are used to quantify expression of a gene [19,76,77,78]. They showed global changes to gene expression levels in rodent models, with particular interest in changes to central nervous system (CNS) tissues. Findings from such transcriptomic studies confirmed that ethanol disrupts different physiological processes depending on the timing of alcohol exposure during gestation. For example, Kleiber et al. [76] observed that adult mice exposed to alcohol during the first-trimester equivalent had aberrant expression of genes related to cell migration and cell specialization, whereas expression of neurotransmission and cellular communication genes were most impacted in those exposed to alcohol in the third trimester [76]. 

In addition to steady-state changes in the epigenome, the way genes behave in response to stressors (i.e., up- or down-regulated), such as inflammation, was shown to persist in adult mice exposed to alcohol in utero [79]. What this means is that individuals with FASD may not have typical responses at the molecular level when responding to their environment later in life, based on the epigenetic programming that occurred during gestation.

Changes to epigenetic modifications are the likely mechanism mediating these changes to the transcriptome. We will focus here on DNA methylation, the best-studied chromatin modification; however, alcohol-induced changes to histone modifications and non-coding RNAs have also been well studied in FASD [19,70].

DNA methylation involves the addition of a methyl group to a cytosine nucleotide, and it occurs at certain locations in the genome (discussed above). Alcohol metabolism can decrease the availability of methyl groups (specifically S-adenosylmethionine (SAM)), which are normally added to cytosine residues by DNA methyltransferase (DNMT) [80]. Alcohol has been shown to inhibit enzymes involved in methionine and SAM production, as well as DNMT itself, thus inhibiting the pathways that facilitate DNA methylation [81,82,83,84,85]. Indeed, initial findings on DNA methylation showed that levels of methylation were reduced in mice exposed to alcohol during gestation [82]. Other findings have since shown that the impact of alcohol on DNA methylation is bidirectional, meaning that it can induce hypomethylation or hypermethylation depending on the specific region, tissue, or loci [19,86,87,88,89]. These studies assessed global levels of DNA methylation in tissues as opposed to specific genes. The takeaway is that these findings support that exposure to alcohol during gestation has the ability to reprogram the epigenome-wide landscape.

There have also been various studies that assess altered epigenesis in candidate genes, which are genes selected based on known physiological maladaptations in FASD. For example, individuals with FASD are often affected by malaligned stress responses and issues with circadian rhythm regulation. Some very promising epigenetic research in FASD relates to regulatory genes within these two physiological systems that were identified due to their susceptibility to alcohol exposure. The gene encoding proopiomelanocortin (POMC) produces peptides involved in regulating the stress response and brain reward system, while the Period 2 (PER2) gene encodes proteins involved in biological clock regulation. Research has shown that levels of Pomc and Per2 proteins are reduced in the hypothalamus of rodents exposed to alcohol in utero [90,91]. Additionally, high levels of alcohol consumption and/or binge drinking in adult humans were associated with DNA hypermethylation of POMC and PER2 genes [92].

Related research by Govorko et al. [93] was the first to demonstrate that epigenetic modifications to POMC could be transmitted across generations in a rodent model. Using Sprague-Dawley rats, they found that fetal alcohol exposure increased methylation in the promoter region of the POMC gene, leading to decreased POMC gene expression and an abnormally high stress response [93]. Interestingly, the effects persisted up to F3 in the male germline but not in the female germline [93]. These data were extended and replicated in a second study using isogenic Fisher rats, confirming that fetal alcohol exposure led to transmissible epigenetic changes in POMC via the male germline, leading to anxiety-like behaviours and stress hyperresponsiveness in the offspring [92]. While the transgenerational effect has not been studied in humans, the Collaborative Initiative on Fetal Alcohol Spectrum Disorders (CIFASD) used DNA obtained from buccal swabs to examine POMC and PER2 methylation patterns in their participants. They observed higher DNA methylation in the POMC and PER2 genes in pregnant females consuming moderate to high levels of alcohol, as well as in children that experience prenatal alcohol exposure [94]. Whether or not these epigenetic signatures persist and could be transmitted to the next generation in humans has not been studied. 

It is important to note that epigenetic changes in individuals with FASD do not necessarily translate to physiological changes. For example, although certain methylation patterns associated with FASD have been shown to alter the transcriptome compared to non-FASD controls, others seem to be neutral, with no effect on gene expression [70]. In addition, while these epigenetic signatures are correlated with FASD, establishing causation due to prenatal alcohol exposure remains challenging, as does ruling out concomitant factors. Furthermore, while epigenome changes from buccal swabs may very well represent the epigenome in other tissues, this cannot be confirmed due to the inability to biopsy certain tissues in human participants. Finally, potential for transgenerational inheritance of FASD-related epigenetic changes in humans remains hypothetical at this time.

## 9. Epigenetic Transmission of Trauma 

It has been hypothesized that children born with FASD are more susceptible to trauma-related cognitive and developmental deficits, and one theory is that prenatal alcohol exposure could alter the epigenome in a way that alters the stress response [6,7]. This remains hypothetical, but evidence from animal models supports an increased stress response in children exposed to alcohol in utero [95], as discussed above. From a social work perspective, it is worthwhile to grasp the research evidence supporting trauma transmission that could be mediated by epigenetic mechanisms. It is also important to note that the research is limited, especially in humans, and in particular as it relates to transgenerational inheritance. We summarise the current evidence here. 

Trauma has been defined by the American Psychiatric Association (APA) in the Diagnostic and Statistical Manual of Mental Disorders, Fifth Edition (DSM-V) under the category Trauma- and Stressor-Related Disorders [96]. The diagnostic criteria for traumatic stress disorders include direct exposure, witnessing or learning about a traumatic event that occurred to a close family member or friend, recurrent, intrusive memories/dreams, dissociative reactions (i.e., flashbacks), and persistent avoidance of stimuli associated with the traumatic event [96]. Early childhood trauma is defined as exposure to traumatic experiences between the ages of birth to six years old [97]. Maltreatment in childhood is especially detrimental during this critical neurodevelopment period in a child’s life and can have particularly damaging consequences [8,98,99]. 

There have been various studies researching whether the effects of trauma, or other adverse life events, in humans can be passed onto future generations. However, evidence in this field is lacking; attributing intergenerational and transgenerational effects to epigenetic transmission (epigenetic signatures inherited with no new exposure to the causative environmental stimuli) is, and will remain, a challenge to ascertain in humans [14].

One limitation is the difficulty teasing out social or cultural transmission, whereby new environmental stimuli produce de novo epigenetic signatures that mimic those of previous generations. In animal models, the environment can be controlled to a large extent, but obviously, this is not possible in humans, and confounding variables limit our ability to study the origin(s) of transmission. This is one reason why there are so many gaps in this area of research. Only when an epigenetic signature is capable of passing through generations can we hypothesize that it is due to the original exposure (assuming the same environmental stimulus is no longer present for progeny). The impact of social transmission is discussed in a later section. 

Another limitation in human studies is often the inability to access specific tissues, such as brain tissue, where many of the epigenetic changes of interest are believed to occur. A study by McGowan et al. [100] did use post-mortem brain tissue from suicide victims to examine epigenetic changes in stress-related genes, similar to the Weaver rat pups that were raised by non-nurturing mothers [61,100]. The glucocorticoid receptor gene, NR3C1, is perhaps one of the best-studied candidates in trauma-related epigenomic programming due to its importance in the hypothalamic-pituitary-adrenal (HPA) stress response. In this study, individuals who had experienced childhood trauma had increased methylation in the promoter region of the neuron-specific NR3C1 gene compared to controls, which correlated with decreased NR3C1 expression [100]. This rare brain tissue study was able to detect a stable epigenetic marker stemming from childhood abuse which lasted to adulthood, within a gene known to be associated with a heightened and prolonged stress response [61,100]. While this study did not show any intergenerational epigenetic transmission of trauma, it did help support potential consistency with animal model findings and showed a stable epigenetic mark from childhood to adulthood. 

The difficulty in obtaining biopsies over multiple generations is obvious, especially when we consider samples from the central nervous system, where arguably trauma-related effects are best studied. As such, the majority of researchers have overcome this disadvantage by using blood samples to study methylation patterns, such as peripheral or cord blood mononuclear cells (BMCs) [101,102]. For example, similar findings to McGowan et al. [100]—higher methylation of the NR3C1 promoter region—have been observed in offspring of mothers exposed to stressors during pregnancy [103,104,105,106]. However, in these cases, peripheral and cord blood cells were used as opposed to CNS tissue. One drawback of BMCs is that many epigenetic changes are tissue-specific, and even cell-specific, so epigenetic changes documented in blood cells may not necessarily be reflective of other tissues [107,108], such as those directly influencing the HPA-stress response in the CNS. Another obstacle related to studying transmission of epigenetic markers is that many of the modifications are genetically programmed and thus relate back to differences in DNA sequence [108]. As such, given that the DNA sequence itself is inherited, this may be the reason for the observed epigenetic state of that gene, as opposed to transmissible influences.

Another proxy used to estimate potential trauma transmission across generations includes assessing low birth weight, cardiovascular health, and hormone levels, which are deemed secondary effects [101,102,109]. For example, studies examining cortisol levels, which is a hormone used to indicate stress, were examined in offspring of Holocaust survivors. It was observed that offspring who were conceived after the Second World War had higher cortisol levels than control subjects [110]. Studies of this nature suggest the possible transmission of certain epigenetic traits that developed from trauma, specifically collective trauma, could be inherited by subsequent generations [110]. To follow up the putative mechanisms mediating transmission of these higher cortisol levels, Yehuda and colleagues examined epigenetic changes in the glucocorticoid receptor, NR3C1, and a related cortisol protein, FKBP5 [102,111,112]. More specifically, they observed that Holocaust survivors had increased methylation at a certain site of the FKBP5 gene compared to controls, whereas their grown children exhibited decreased methylation at the same FKBP5 site compared to controls [102,111]. In other words, Holocaust survivors and their offspring exhibited differences in methylation patterns of cortisol-related genes. This evidence supports the role of epigenetic mechanisms in intergenerational transmission of trauma effects. At the same time, we also cannot exclude potential effects of social transmission.

A well-known example of environmental adversity transmission includes studies related to the Dutch famine, which took place between 1944 and 1945 during the Second World War in Holland [101,113,114]. A food embargo was placed on Western Holland by the German occupying forces, which ultimately cut food rations down to approximately 400–800 calories/day [115]. This widespread famine affected the offspring of the women who were pregnant during that time. Depending on how far along in gestation the mother was, offspring exposed to malnourishment in utero were found to have a higher prevalence of cardiovascular disease, obesity, low birth weight, diabetes, and breast cancer [101,113,114]. It has also been proposed that anxiety related to the events (war, extreme cold, famine) may have at least in part mediated these effects, given the parallels in later-life effects associated with prenatal under-nutrition or prenatal stress [114]. 

Epigenetic transmission is one proposed mechanism to explain this intergenerational impact of prenatal famine exposure on poor health outcomes later in life. The Dutch Hunger Winter families study showed that in utero adverse events altered DNA methylation, with sex-specific differences noted, as well as impact of gestational stage at exposure [116,117]. For example, prenatal exposure to famine decreased DNA methylation on the imprinted gene encoding the insulin-like growth factor 2 (IGF2) when compared to non-exposed siblings [116]. This hypomethylation of IGF2 persisted over six decades [116]. Studies of seasonal under-nutrition in Gambia also support the impact of maternal diet influence on DNA methylation patterns that persists into adulthood [108]. In contrast, epigenome analysis by Veenendaal et al. [118] failed to show any differences in DNA methylation patterns between the famine cases (exposed) and controls (not exposed) in the candidate genes that they studied.

In addition to the prenatal maternal stress impact on the HPA axis, it is also considered to be a source of altered immune system functioning for the fetus [119]. Another study examining in utero adverse experiences assessed pregnant mothers who had experienced the Quebec ice storm in 1998 [109]. The ice storm downed power lines that created power outages which lasted from 3 h up to 6 weeks in some regions of the province, and women reported experiencing high levels of stress during pregnancy. They measured DNA methylation in T-cells (cells involved in the immune system) of offspring at 8yr and 13yr to determine if the mother’s perceived stress from the natural disaster correlated with increased methylation patterns in T-cells [109]. Hypermethylation of specific T-cell genes was associated with higher levels of stress [109]. These findings draw parallels to the increased inflammatory immune response seen in individuals who have been exposed to maltreatment as children [120]. 

The challenge in measuring secondary effects lies in the inability to identify causation between the environmental stimulus and the epigenetic marker; these types of studies are able to demonstrate correlation without finding the precise mechanism involved. This creates a grey area in the field of epigenetics, which has caused some scientists to advise caution in interpreting conclusions [53,121,122]. Sometimes, the media has even shown restraint, noting that there is a debate in science surrounding epigenetics [123], although often, popular media fails to explain the limitations of the evidence. 

Reproducible experimental data on true transgenerational epigenetic transmission come from rodent studies and are limited. The studies discussed above provide evidence to suggest that this process may be similar in humans. However, prospective longitudinal studies are required to understand whether these effects are real, and there are obvious difficulties in recruiting and following human cohorts due to our long lifespan and long reproductive cycle. Making definitive conclusions about transgenerational transmission of trauma resulting from transmissible epigenetic changes through the germline remains premature at this time [49]. In addition, confounding effects of social transmission make it difficult to tease out potential impact of biological effects versus new environmentally induced epigenetic changes.

## 10. Social Transmission of Trauma 

The word “transmission” does not automatically imply that phenotypes persisting across generations are due to biological inheritance. When considering transmission of trauma effects in FASD, a discussion of social transmission pathways is equally as, if not more, important. 

Environmental adversity, such as trauma, can also be transmitted through the behavioural patterns arising from cultural, familial, parenting, and other forms of social relational social environments. Individuals with unresolved trauma continue to express that in their interactions with others. As a parent or grandparent, unresolved trauma influences how a child comes to understand their place in the family as well as larger society [124]. Social pathways can be thought of as how a person becomes socialized into the cultural or group experience of a traumatic history as well as into family. It also acts as a way for a person to understand intimate relationships. 

Fitzgerald, London-Johnson, and Gallus [124] found that trauma in women increased the negative qualities of relationships and decreased positive ones. FASD and trauma act in compounding and intersecting ways that serve to increase the probabilities of intergenerational social transmission of trauma [5,6]. There is also a clear link between those with FASD and elevated adverse childhood event (ACE) scores, which further entrenches the presence of trauma arising from inter-generational social transmission [5].

**Figure 3:**
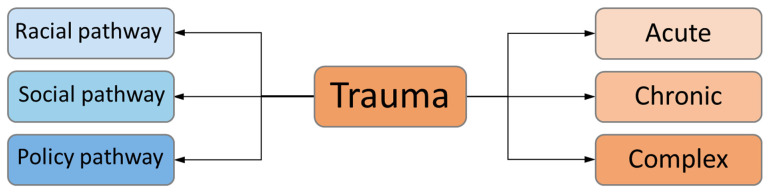
Intersecting pathways of transmission reinforce different types of trauma experiences.

Social transmission is reinforced through intersecting pathways as seen in Figure 3. On the left-hand side, the three pathways of social, policy, and race intersect with the presence of trauma. Social pathways include the family systems but also community systems that may see a person with trauma, especially when expressing FASD behaviours, as problematic and disruptive. Policies, such as child intervention, education, health, and criminal justice act as ways in which disability is given prominence as opposed to strength [38]. The racial pathway box serves as a reminder that child welfare, justice, and related systems are far more likely to be involved with the racialized population [125,126,127]. In addition, trauma does not have a single expression but rather can range from acute to chronic presentations and may be complex. This can have a profound impact on the intersection with the pathways on the right and left sides of Figure 3 (see, for example, [128]). 

A substantial body of literature shows that the developmental trajectory of children into adulthood, and onwards, is adversely affected by exposure to what has been commonly referred to as adverse childhood experiences (ACE) [129]. These have been shown to impact long-term mental, emotional, relational, and physical health over the lifetime of individuals [130]. Resulting behavioural patterns can therefore propagate these effects of trauma onto the next generation through interactions with children and grandchildren. There is research showing a compounding impact of FASD and ACE experiences [131]. Speaking of the impacts of ACEs, Price et al. [6] noted, “Traumatic childhood experiences (trauma) such as maltreatment can lead to markedly similar neurological, cognitive and behavioral deficits as those caused by PAE” (p. 90). 

Griffin [132] has added to the question of trauma at the individual level across generations stating, “Trauma is defined by the presence of three factors: events, experiences and effects. The event is the objective adverse incident that produces toxic stress” (p. S279) while noting that there is also an individual subjective response to the event. Griffin [132] goes on to acknowledge that individual differences in genetics, neurodevelopment, overall health, and resilience, as well as the presence of protective factors in the person’s life, will have an effect on transmission. We can appreciate how understanding the impact of trauma on an individual is complex and that further complexity is added with reinforcement or transmission through various social, racial, and policy pathways. 

One classic example of social transmission of trauma and modeling these pathways comes from the colonial assimilation policies of Indigenous peoples in Canada and how this trauma impacted multiple generations [133]. This includes the higher prevalence of FASD found in Indigenous populations compared to non-Indigenous populations [3]. Trauma that occurs in one generation arising from assimilation activities is carried over into the subsequent generations through various relational patterns and becomes reinforced through various environmental impacts. Parenting models are lost over generations, and child rearing occurs from a place of trauma. Substance abuse is often sought as a coping mechanism, which tends to extend FASD into the subsequent generations. If the healing from the trauma does not occur in the first or subsequent generations, then the social relational transmission will continue. In addition, there may be compounding effects as new trauma is added upon pre-existing effects from the prior generations. Further, trauma-based coping becomes entrenched, including alcohol as a coping mechanism, such that each prior generation is modeling and teaching trauma-based relational skills.

Furthermore, if we focus on the impact of the Indian Residential Schools (IRS) in Canada, we can see how trauma moved across multiple generations causing long-term damage. Imagine the scenario (well documented in the reports of the Truth and Reconciliation Commission of Canada [133]) of virtually all of the children being removed from a community to be sent off to the IRS. One can imagine the hole that is left in the community as silence replaces the voices of children. Furthermore, the majority of those children will not return, and those that do are very traumatized [133]. The trauma did not stop with the closure of the last IRS in 1996. Rather, as a result of Canada’s ongoing policies to forcibly assimilate Indigenous peoples, Indigenous children have, since the 1950s, been brought into care of child welfare at rates far in excess of their proportion of the national population [133,134]. Higher rates of childhood abuse have been observed in the Indigenous community [135]. Adults who reported being victims of maltreatment as children were more likely to report use of illegal drugs and binge drinking and experienced homelessness compared to non-victims in the same age group [135]. The higher rates in Indigenous communities likely result from lacking social determinants of health [136]. This takes us back to Figure 3, which shows how the social, racial, and policy pathways in transmission of trauma have been well documented.

## 11. Challenge of Discerning Social and Epigenetic Transmission 

In social work, perhaps one of the most vexing questions that needs to be addressed is finding a way to define how trauma affects an individual using a scientific lens. In a review of epigenetics and PTSD, Ryan et al. [137] noted how trauma during critical periods of development in utero was more likely to have significant long-term impacts, since these periods coincide with epigenetic programming. As discussed in Section 9, evidence supports that early-life trauma can impact epigenetic modifications in both the CNS and peripheral tissues [100,103,104,105,106]. Youssef et al. [138] reviewed a growing body of evidence for transmission of trauma from a traumatized parent to the next generation (intergenerational); however, much of the evidence supports epigenetic changes due to direct environmental exposures as opposed to inheritance. 

Matthews and Phillips [139] emphasize the social implications of being able to establish the linkages, noting, “the fact that prenatal stress can lead to transgenerational effects on stress physiology and behaviors may have important societal consequences, as it could provide a biological explanation for the generational persistence of human behaviours in people exposed to adversity” (p. 100). In other words, we see how behaviours or physiological consequences (phenotypes) resulting from trauma can persist across multiple generations. 

Importantly, the difficulty remains in how to discern if a particular phenotype was moving across generations due to epigenetic effects as opposed to social transmission, or both. Currently, a feasible approach to tackle this question does not exist, and further research is needed. We are limited by the challenge of teasing out cultural, policy, relational, and behavioural impacts, whereby new environmental stimuli produce de novo epigenetic signatures that mimic those of previous generations. Yet, it remains an important question as the answer could certainly influence interventions in social work and other related areas. 

Attributing the transmission to epigenetic inheritance will remain a challenge in humans, as we are unable to control the environment, such as ensuring no new exposure to the causative environmental stimuli [14]. As such, we are very much lacking research and evidence in humans. The best evidence remains in rodent models where the environment can be controlled to a certain extent, helping to distinguish biological versus social transmission. In addition, animal studies allow access to CNS tissues, such as specific parts of the brain, which has clear challenges in humans. Designing prospective longitudinal studies in human cohorts, which include tissue sampling across multiple generations, would be ideal for studying epigenetic inheritance. As discussed, this has obvious barriers, including high costs, subject retention, and unmanageable study duration.

**Figure 4:**
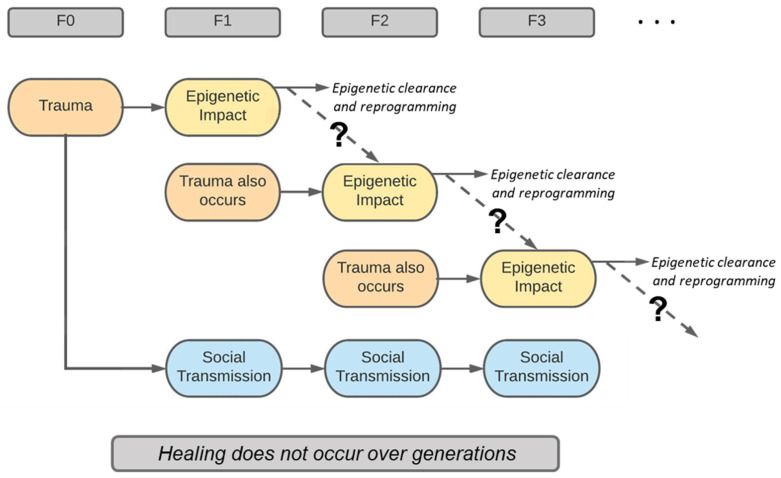
Proposed model of how social and biological transmission of trauma may overlap. In the absence of healing interventions that alter the social environment, trauma will re-occur in each new generation, leading to social transmission (blue). Epigenetic changes due to trauma may persist into adulthood, but these are cleared away during embryonic reprogramming. At the present time, there is no known mechanism for escaping epigenetic clearance, and thus the inheritance of epigenetic marks across generations in humans remains hypothetical (represented by question mark). F—filial.

Science continues to examine whether effects of adverse life events in humans can be passed on to future generations biologically (Section 9). The current understanding of epigenetics is that the parental epigenomes (apart from imprinted genes) are erased and reprogrammed during human embryonic development (Section 6 and Figure 4). There is no currently understood mechanism as to how a trauma-imposed epigenetic signature could be transmitted through the germline to the next generation, thereby avoiding this clearance/reprogramming phase (Figure 4). If consistent epigenetic signatures are witnessed across generations, the challenge remains distinguishing whether the epigenetic marks were inherited via the germline versus the same marks developing de novo in the offspring (Figure 4). As such, the concept of true transgenerational epigenetic inheritance remains a controversial hypothesis [14,53,66]. 

At present, we lack the evidence necessary to affirm *transgenerational* transmission related to trauma and FASD via epigenetic mechanisms. Should future research determine that true germline epigenetic transmission occurs across multiple generations, then the nature of interventions may need to adapt. Professionally, the more that social workers can support those with FASD to make choices about ways to reduce risk and improve healing in their own generation, the more that healthier outcomes can be expected in the next generation.

## 12. Why Is This Important to Social Work? 

Interventions to prevent FASD, as well as to minimize frequency and support mothers at risk and those impacted by the disorder, are important to reduce both frequency and impact. In many regions, social work is often called upon to work with this population in the diagnostic, support, and prevention environments. An understanding of how trauma transmission may or may not be related to epigenetics is important in social work, in order to better understand how interventions can be structured. If we do not address the intergenerational effects, we are destined to propagate traumatic impacts. If further research affirms the impact of epigenetics, we can begin to think about complex solutions that are not open to us at this time. 

Social work, as a profession, has paid a great deal of attention to the social, policy, familial, community, and cultural pathways of trauma and its transmission. Social determinants of health (SDH) have been a significant focus in that families and communities at risk appear to be held in positions of economic, education, health, housing, and service deprivations over time. The notion in social work has been that the SDH needs to be changed if populations are going to attain self-determination and the hope of being released from these intergenerational patterns. Maylea [140] argues that social work has not been effective in altering the pattern of SDH which, in turn, sustains social transmission of trauma (Figure 4). We suggest that this may be the case in much of the social contexts of FASD and other traumatized populations. In other words, social work serves systems of government and society that are not addressing SDH in meaningful ways. Without deliberative focus on changing the social conditions in which FASD and trauma link from generation to generation, social work is destined to be a sustaining force, rather than a healing force. More research is needed as the epigenetics story may act as a way for us to think about the importance of designing interventions across generations.

Other voices within social work see promise in coming to understand that trauma can be passed across generations through epigenetics. For example, Combs-Orme [141] states, “Epigenesis provides a basis for understanding why poor and minority populations continue to lag behind in our nation, as it gives us an understanding of the mechanisms through which poverty, oppression, and disadvantage exercise their effects on physiology” (p. 7). However, others raise significant caution about being too quick to see epigenetics and science as the way for social work to understand how trauma moves across generations [121,142,143]. Juengst et al. (2014) question the ethics of the current dialogues that over-promise what epigenetics can really tell us about the impact of trauma over generations [121]. We agree that although it is an attractive hypothesis, we do not yet have the evidence base to support a mechanism for understanding how social factors influence epigenetic inheritance across multiple generations. 

Epigenetics could potentially offer pathways to build effective interventions that ameliorate the impacts of trauma and stress [142], and future research may one day support this goal. In the short term, we postulate that SDH improvements are likely to support the next generation at both the social and epigenetic levels, improving the pathways to healthier next generations.

Lappé [144] reports that the public media dialogue reinforces messages to individuals who intersect with social work that not only indicate broad implications from epigenetics but also quite specific statements about the impact on having and raising children. There is a potential significant ethical question in this work. Epigenetic theory is not meant to be interpreted as a reason for one generation to not have children, nor does evidence support any related arguments. There exists a potential for individuals to make family planning decisions based on perceived epigenetic impacts, which are not yet evidence-based. Rather, social workers can ethically help clients with FASD to understand the linkages between trauma, coping, and preparing for their role as a parent should they have children. We raise this so as to emphatically support preparing one generation to most effectively enter into the role of parenthood. These types of practices are needed to help support healing.

There is good rationale for social work to support families in understanding the impact of epigenetics in both inter- and transgenerational trauma transmission in FASD. While epigenetics represents an exciting opportunity to potentially better understand trauma within/across generations, the evidence is both limited and prone to misinterpretations [142,145]. Of significance, when completing a literature search of biomedical and life sciences publications (PubMed and Medline) using the terms “trauma”, “epigenetics”, and “FASD”, there are no results, highlighting the lack of current evidence. Outside of FASD, the data supporting epigenetic transmission of trauma in humans are limited (Section 9). Furthermore, in the field of social work, it can be difficult to know which biomedical resources are credible, and providers must be careful not to make assumptions about biological transmission and/or extrapolating evidence too far. Extrapolating findings from inconclusive research could be potentially harmful in framing social work policy. In other words, understanding what science actually supports in terms of modeling epigenetic trauma transmission and what is not yet evidence-based, is of importance. 

With limited resources, we believe it is important to target interventions where the most benefit stands to be gained. While future therapeutic interventions may one day target changes to the epigenome, the development of epidrugs (drugs that target DNA methylation and histone acetylation) to treat psychological trauma remains in its infancy [146]. Given the impact of social transmission, targeting the social determinants of health would likely have a more far-reaching impact. This includes strategies for healing that address intergenerational relational pathways related to substance use [147]. Furthermore, it is probable that focusing on SDH could also improve any biological impacts. 

While epigenetic mechanisms may ultimately hold promise for future interventions and prevention strategies, we caution against an over-interpretation of the actual evidence and that extending conclusions beyond what has actually been observed experimentally is not advisable in framing current policy and practice. Indeed, we agree with the conclusions of White and Wastell (2016) that social work may do harm by embracing science that is not yet to a point that should be guiding decisions about the lives of clients [142]. 

We have explained why we feel that a better understanding in the application of current scientific research in social work is needed. We are equally of the view that science needs to continue investigating the possible impacts of epigenetics in inter- and transgenerational trauma, and social work needs to help define research priorities that could impact practice and ethical decisions. Perhaps the most benefit will come from interdisciplinary approaches where social work helps to inform the science and likewise science helps to inform the social work.

## 13. Conclusions

We have summarized and connected current research in both the sciences and social sciences to map out which parts of the trauma transmission pathways in FASD can be supported by scientific evidence. We presented theoretical models of transmission that represent how biological and social transmission overlap. Finally, we noted the importance of understanding the science behind potential epigenetic transmission and what is actually evidence-based when developing social work policy and interventions in FASD. Currently, we do not have enough research to discern which parts of inter- and transgenerational trauma transmission are social versus biological, and we argue that it is premature to place too much emphasis on epigenetics, without the appropriate evidence base. Should the molecular biology research advance to support these, then we could start developing new ways of providing cross-generational support and programming. 

Results from a simple Google search are enough to witness the excitement and draw associated with transgenerational epigenetic inheritance. In FASD, indeed, it provides a potential mechanism to help explain why we may see certain trauma-related physiological phenotypes from one generation to the next, outside of those that can be directly linked to prenatal alcohol exposure. Epigenetic control represents an extremely complicated diverse layer of regulation in how our genes are expressed, one in which we are only beginning to understand the degree of complexity involved. 

While this field represents an exciting opportunity for potentially better understanding trauma across generations, it is important to understand that it is very prone to errors, misrepresentation, and misinterpretations [145]. Extrapolating findings from inconclusive research can be potentially harmful in framing social work policy. With limited resources, it is important to target interventions where the most benefit stands to be gained. While future therapeutic interventions may one day target changes to the epigenome, the development of epidrugs to treat psychological trauma is not yet a reality. Given the impact of social transmission, targeting the social determinants of health would likely have a more far-reaching impact. In addition, over-attributing behaviours to epigenetics may provide barriers to those seeking treatment if they feel that epigenetics is something that cannot be surmounted. 

Reproducible experimental data supporting true transgenerational epigenetic transmission are limited and come from non-human studies. The process may be similar in humans; however, prospective longitudinal studies would be needed to understand whether these effects are real, and there are obvious challenges in recruiting and following human cohorts for such an extensive length of time due to our long lifespan and long reproductive cycle. In addition, controlling for confounding external variables, such as behavioural and cultural transmission, makes it near impossible to tease out true germline heritable transmission. Because many subjects would be lost to follow-up, combined with potential subtle endpoint effects, the starting sample size would need to be extremely large. For these reasons, as well as unreasonable costs, designing lengthy longitudinal human studies remains unlikely at this time. Therefore, making definitive conclusions about transgenerational transmission of trauma within (and outside of) the FASD population resulting from transmissible epigenetic changes through the germline remains premature at this time [49]. 

As the field continues to grow and new mechanisms to study the epigenome evolve, we may see support for various hypothetical models. Nonetheless, it will always remain very challenging to fully separate effects of biological versus social transmission and/or compounding effects. As such, it is important that those involved in social work understand the evidence, or lack thereof, when it comes to what exactly research can support with respect to epigenetic-based transmission of trauma across generations. While epigenetic mechanisms may ultimately hold promise for future interventions and prevention strategies, caution is required if social work is to embrace epigenetic models in framing current policy and practice. Building on best practices and the evidence base remains the most effective approach as scientific research continues to uncover the mechanisms of epigenetic transmission.

## Data Availability

Not applicable.

## References

[1] Tan C.H., Denny C.H., Cheal N.E., Sniezek J.E., Kanny D. (2015). Alcohol Use and Binge Drinking among Women of Childbearing Age—United States, 2011–2013. MMWR Morb. Mortal. Wkly. Rep..

[2] Popova S., Lange S., Probst C., Gmel G., Rehm J. (2017). Estimation of National, Regional, and Global Prevalence of Alcohol Use during Pregnancy and Fetal Alcohol Syndrome: A Systematic Review and Meta-Analysis. Lancet Glob. Health.

[3] Popova S., Lange S., Shield K., Burd L., Rehm J. (2019). Prevalence of Fetal Alcohol Spectrum Disorder among Special Subpopulations: A Systematic Review and Meta-Analysis. Addiction.

[4] Public Health Agency of Canada Report on the State of Public Health in Canada 2010. https://www.canada.ca/en/public-health/corporate/publications/chief-public-health-officer-reports-state-public-health-canada/addressing-stigma-toward-more-inclusive-health-system.html.

[5] Flannigan K., Kapasi A., Pei J., Murdoch I., Andrew G., Rasmussen C. (2021). Characterizing Adverse Childhood Experiences among Children and Adolescents with Prenatal Alcohol Exposure and Fetal Alcohol Spectrum Disorder. Child Abus. Negl..

[6] Price A., Cook P.A., Norgate S., Mukherjee R. (2017). Prenatal Alcohol Exposure and Traumatic Childhood Experiences: A Systematic Review. Neurosci. Biobehav. Rev..

[7] Lam V.Y.Y., Raineki C., Takeuchi L.E., Ellis L., Woodward T.S., Weinberg J. (2018). Chronic Stress Alters Behavior in the Forced Swim Test and Underlying Neural Activity in Animals Exposed to Alcohol Prenatally: Sex- and Time-Dependent Effects. Front. Behav. Neurosci..

[8] Anda R.F., Felitti V.J., Bremner J.D., Walker J.D., Whitfield C., Perry B.D., Dube S.R., Giles W.H. (2006). The Enduring Effects of Abuse and Related Adverse Experiences in Childhood: A Convergence of Evidence from Neurobiology and Epidemiology. Eur. Arch. Psychiatry Clin. Neurosci..

[9] Hyter Y.D. (2012). Complex Trauma and Prenatal Alcohol Exposure: Clinical Implications. Perspect. Sch. Issues.

[10] Piras F., Vecchio D., Assogna F., Pellicano C., Ciullo V., Banaj N., Edden R.A.E., Pontieri F.E., Piras F., Spalletta G. (2021). Cerebellar Gaba Levels and Cognitive Interference in Parkinson’s Disease and Healthy Comparators. J. Pers. Med..

[11] Blumenthal H., Blanchard L., Feldner M., Babson K., Leen-Feldner E., Dixon L. (2008). Traumatic Event Exposure, Posttraumatic Stress, and Substance Use Among Youth: A Critical Review of the Empirical Literature. Curr. Psychiatry Rev..

[12] Jaenisch R., Bird A. (2003). Epigenetic Regulation of Gene Expression: How the Genome Integrates Intrinsic and Environmental Signals. Nat. Genet..

[13] Chudley A.E., Riley E.P., Clarren S., Weinberg J., Jonsson E. (2011). Genetic Factors Contributing to FASD. Fetal Alcohol Spectrum Disorder: Management and Policy Perspectives of FASD.

[14] Yehuda R., Lehrner A. (2018). Intergenerational Transmission of Trauma Effects: Putative Role of Epigenetic Mechanisms. World Psychiatry.

[15] Mead E.A., Sarkar D.K. (2014). Fetal Alcohol Spectrum Disorders and Their Transmission through Genetic and Epigenetic Mechanisms. Front. Genet..

[16] Henriques M. Can the Legacy of Trauma Be Passed down the Generations?. https://www.bbc.com/future/article/20190326-what-is-epigenetics.

[17] DeAngelis T. (2019). The Legacy of Trauma. Am. Psychol. Assoc..

[18] Gupta K.K., Gupta V.K., Shirasaka T. (2016). An Update on Fetal Alcohol Syndrome—Pathogenesis, Risks, and Treatment. Alcohol. Clin. Exp. Res..

[19] Ungerer M., Knezovich J., Ramsay M. (2013). In Utero Alcohol Exposure, Epigenetic Changes, and Their Consequences. Alcohol Res. Curr. Rev..

[20] Young J.K., Giesbrecht H.E., Eskin M.N., Aliani M., Suh M. (2014). Nutrition Implications for Fetal Alcohol Spectrum Disorder. Adv. Nutr..

[21] O’Malley K.D., Rich S.D. (2013). Clinical Implications of a Link Between Fetal Alcohol Spectrum Disorders (FASD) and Autism or Asperger’s Disorder—A Neurodevelopmental Frame for Helping Understanding and Management. Intech.

[22] Jones K.I., Smith D.W., Ulleland C.N., Pykowicz Streissguth A. (1973). Pattern of Malformation in Offspring of Chronic Alcoholic Mothers. Lancet.

[23] Himmelreich M., Lutke C.J., Travis Hargrove E., Begun A.L., Murray M.M. (2020). Lay of the Land: Fetal Alcohol Spectrum Disorder (FASD) as a Whole Body Diagnosis. The Routledge Handbook of Social Work and Addictive Behaviors.

[24] Burd L., Martsolf J.T., Klug M.G., Kerbeshian J. (2003). Diagnosis of FAS: A Comparison of the Fetal Alcohol Syndrome Diagnostic Checklist and the Institute of Medicine Criteria for Fetal Alcohol Syndrome. Neurotoxicol. Teratol..

[25] O’Leary C. (2004). Fetal Alcohol Syndrome: Diagnosis, Epidemiology, and Developmental Outcomes. J. Paediatr. Child Health.

[26] Doyle L.R., Mattson S.N. (2015). Neurobehavioral Disorder Associated with Prenatal Alcohol Exposure (ND-PAE): Review of Evidence and Guidelines for Assessment. Curr. Dev. Disord. Rep..

[27] Lynch M.E., Kable J.A., Coles C.D. (2015). Prenatal Alcohol Exposure, Adaptive Function, and Enty into Adult Roles in Prospective Study of Young Adults. Neurotoxicol. Teratol..

[28] Moore E.M., Riley E.P. (2015). What Happens When Children with Fetal Alcohol Spectrum Disorders Become Adults?. Curr. Dev. Disord. Rep..

[29] Panczakiewicz A.L., Glass L., Coles C.D., Kable J.A., Sowell E. (2016). lizabet. R.; Wozniak, J.R.; Jones, K.L.; Riley, E.P.; Mattson, S.N. Neurobehavioral Deficits Consistent across Age and Sex in Youth with Prenatal Alcohol Exposure. Alcohol. Clin. Exp. Res..

[30] Carter C.R., Jacobson J.L., Molteno C.D., Dodge N.C., Meintjes E.M., Jacobson S.W. (2016). Fetal Alcohol Growth Restriction and Cognitive Impairment. Pediatrics.

[31] May P.A., Chambers C.D., Kalberg W.O., Zellner J., Feldman H., Buckley D., Kopald D., Hasken J.M., Xu R., Honerkamp-Smith G. (2018). Prevalence of Fetal Alcohol Spectrum Disorders in 4 US Communities. JAMA-J. Am. Med. Assoc..

[32] Coles C.D. (2011). Discriminating the Effects of Prenatal Alcohol Exposure from Other Behavioral and Learning Disorders. Alcohol Res. Health.

[33] Stevens S.A., Nash K., Koren G., Rovet J. (2013). Autism Characteristics in Children with Fetal Alcohol Spectrum Disorders. Child Neuropsychol..

[34] McLachlan K., Flannigan K., Temple V., Unsworth K., Cook J.L. (2020). Difficulties in Daily Living Experienced by Adolescents, Transition-Aged Youth, and Adults with Fetal Alcohol Spectrum Disorder. Alcohol. Clin. Exp. Res..

[35] Temple V., Cook J.L., Unsworth K., Roberts N. (2021). Prenatal Alcohol Exposure and Autism Spectrum Disorder in 39 Children and Adults: Examination of Behavioural and Cognitive Profiles. J. Ment. Health Res. Intellect. Disabil..

[36] Mukherjee R., Layton M., Yacoub E., Turk J. (2011). Autism and Autistic Traits in People Exposed to Heavy Prenatal Alcohol: Data from a Clinical Series of 21 Individuals and Nested Case Control Study. Adv. Ment. Health Intellect. Disabil..

[37] Bishop S., Gahagan S., Lord C. (2007). Re-Examining the Core Features of Autism: A Comparison of Autism Spectrum Disorder and Fetal Alcohol Spectrum Disorder. J. Child Psychol. Psychiatry Allied Discip..

[38] Choate P., Badry D. (2019). Stigma as a Dominant Discourse in Fetal Alcohol Spectrum Disorder. Adv. Dual Diagn..

[39] Barker C., Kulyk J., Knorr L., Brenna B. (2011). Open Inclusion or Shameful Secret: A Comparison of Characters with Fetal Alcohol Spectrum Disorders (FASD) and Characters with Autism Spectrum Disorders (ASD) in a North American Sample of Books for Children and Young Adults. Int. J. Spec. Educ..

[40] Lange S., Probst C., Gmel G., Rehm J., Burd L., Popova S. (2017). Estimation of National, Regional, and Global Prevalence of Fetal Alcohol Spectrum Disorder among Children and Youth: A Systematic Review and Meta-Analysis. JAMA Pediatr..

[41] Chasnoff I.J., Wells A.M., King L. (2015). Misdiagnosis and Missed Diagnoses in Foster and Adopted Children with Prenatal Alcohol Exposure. Pediatrics.

[42] Berger S.L., Kouzarides T., Shiekhattar R., Shilatifard A. (2009). An Operational Definition of Epigenetics. Genes Dev..

[43] Waddington C.H. (1942). Canalization of Development and the Inheritance of Acquired Characters. Nature.

[44] Deichmann U. (2016). Epigenetics: The Origins and Evolution of a Fashionable Topic. Dev. Biol..

[45] Peschansky V.J., Wahlestedt C. (2014). Non-Coding RNAs as Direct and Indirect Modulators of Epigenetic Regulation. Epigenetics.

[46] Deichmann U. (2020). The Social Construction of the Social Epigenome and the Larger Biological Context. Epigenet. Chromatin.

[47] Pickersgill M., Niewöhner J., Müller R., Martin P., Cunningham-Burley S. (2013). Mapping the New Molecular Landscape: Social Dimensions of Epigenetics. New Genet. Soc..

[48] Norouzitallab P., Baruah K., Vanrompay D., Bossier P. (2019). Can Epigenetics Translate Environmental Cues into Phenotypes?. Sci. Total Environ..

[49] Van Otterdijk S.D., Michels K.B. (2016). Transgenerational Epigenetic Inheritance in Mammals: How Good Is the Evidence?. FASEB J..

[50] Annunziato A. (2008). DNA Packaging: Nucleosomes and Chromatin|Learn Science at Scitable. Nat. Educ..

[51] Edwards J.R., Yarychkivska O., Boulard M., Bestor T.H. (2017). DNA Methylation and DNA Methyltransferases. Epigenet. Chromatin.

[52] Statello L., Guo C.J., Chen L.L., Huarte M. (2021). Gene Regulation by Long Non-Coding RNAs and Its Biological Functions. Nat. Rev. Mol. Cell Biol..

[53] Horsthemke B. (2018). A Critical View on Transgenerational Epigenetic Inheritance in Humans. Nat. Commun..

[54] Perez M.F., Lehner B. (2019). Intergenerational and Transgenerational Epigenetic Inheritance in Animals. Nat. Cell Biol..

[55] Cedar H., Bergman Y. (2009). Linking DNA Methylation and Histone Modification: Patterns and Paradigms. Nat. Rev. Genet..

[56] Seisenberger S., Peat J.R., Hore T.A., Santos F., Dean W., Reik W. (2013). Reprogramming DNA Methylation in the Mammalian Life Cycle: Building and Breaking Epigenetic Barriers. Philos. Trans. R. Soc. B Biol. Sci..

[57] Neidhart M. (2015). DNA Methylation and Complex Human Disease.

[58] Dolinoy D.C., Huang D., Jirtle R.L. (2007). Maternal Nutrient Supplementation Counteracts Bisphenol A-Induced DNA Hypomethylation in Early Development. Proc. Natl. Acad. Sci. USA.

[59] Kubsad D., Nilsson E.E., King S.E., Sadler-Riggleman I., Beck D., Skinner M.K. (2019). Assessment of Glyphosate Induced Epigenetic Transgenerational Inheritance of Pathologies and Sperm Epimutations: Generational Toxicology. Sci. Rep..

[60] Meaney M.J. (2001). Maternal Care, Gene Expression, and the Transmission of Individual Differences in Stress Reactivity across Generations. Annu. Rev. Neurosci..

[61] Weaver I.C.G., Cervoni N., Champagne F.A., D’Alessio A.C., Sharma S., Seckl J.R., Dymov S., Szyf M., Meaney M.J. (2004). Epigenetic Programming by Maternal Behavior. Nat. Neurosci..

[62] National Center for Biotechnology Information. https://www.ncbi.nlm.nih.gov.

[63] Morgan H.D., Sutherland H.G.E., Martin D.I.K., Whitelaw E. (1999). Epigenetic Inheritance at the Agouti Locus in the Mouse. Nat. Genet..

[64] Lu D., Willard D., Patel I.R., Kadwell S., Overton L., Kost T., Luther M. (1994). Agouti Protein Is an Antagonist of the Melanocyte-Stimulating-Hormone Receptor. Nature.

[65] Pang T.Y., Short A.K., Bredy T.W., Hannan A.J. (2017). Transgenerational Paternal Transmission of Acquired Traits: Stress-Induced Modification of the Sperm Regulatory Transcriptome and Offspring Phenotypes. Curr. Opin. Behav. Sci..

[66] Heard E., Martienssen R.A. (2014). Transgenerational Epigenetic Inheritance: Myths and Mechanisms. Cell.

[67] Lehrner A., Yehuda R. (2018). Cultural Trauma and Epigenetic Inheritance. Dev. Psychopathol..

[68] Brink R.A. (1956). A Genetic Change Associated with the R Locus in Maize Which Is Directed and Potentially Reversible. Genetics.

[69] Coe E.H. (1959). A Regular and Continuing Conversion-Type Phenomenon at the B Locus in Maize. Proc. Natl. Acad. Sci. USA.

[70] Lussier A.A., Weinberg J., Kobor M.S. (2017). Epigenetics Studies of Fetal Alcohol Spectrum Disorder: Where Are We Now?. Epigenomics.

[71] Hanson M.A., Gluckman P.D. (2008). Developmental Origins of Health and Disease: New Insights. Basic Clin. Pharmacol. Toxicol..

[72] Herringa R.J. (2017). Trauma, PTSD, and the Developing Brain. Curr. Psychiatry Rep..

[73] Lossie A.C., Muir W.M., Lo C.-L., Timm F., Liu Y., Gray W., Zhou F.C. (2014). Implications of Genomic Signatures in the Differential Vulnerability to Fetal Alcohol Exposure in C57BL/6 and DBA/2 Mice. Front. Genet..

[74] Mason S., Zhou F.C. (2015). Genetics and Epigenetics of Fetal Alcohol Spectrum Disorders. Front. Genet..

[75] Hoyme E.H., Kalberg W.O., Elliott A.J., Blankenship J., Buckley D., Marais A.-S., Manning M.A. (2016). Updated Clinical Guidelines for Diagnosing Fetal Alcohol Spectrum Disorders. Pediatrics.

[76] Kleiber M.L., Mantha K., Stringer R.L., Singh S.M. (2013). Neurodevelopmental Alcohol Exposure Elicits Long-Term Changes to Gene Expression That Alter Distinct Molecular Pathways Dependent on Timing of Exposure. J. Neurodev. Disord..

[77] Hard M.L., Abdolell M., Robinson B.H., Koren G. (2005). Gene-Expression Analysis after Alcohol Exposure in the Developing Mouse. J. Lab. Clin. Med..

[78] Hashimoto-Torii K., Imamura Kawasawa Y., Kuhn A., Rakic P. (2011). Combined Transcriptome Analysis of Fetal Human and Mouse Cerebral Cortex Exposed to Alcohol. Proc. Natl. Acad. Sci. USA.

[79] Lussier A.A., Stepien K.A., Neumann S.M., Pavlidis P., Kobor M.S., Weinberg J. (2015). Prenatal Alcohol Exposure Alters Steady-State and Activated Gene Expression in the Adult Rat Brain. Alcohol. Clin. Exp. Res..

[80] Resendiz M., Chen Y., Oztürk N.C., Zhou F.C. (2013). Epigenetic Medicine and Fetal Alcohol Spectrum Disorders. Epigenomics.

[81] Lu S.C., Huang Z.Z., Yang H., Mato J.M., Avila M.A., Tsukamoto H. (2000). Changes in Methionine Adenosyltransferase and S-Adenosylmethionine Homeostasis in Alcoholic Rat Liver. Am. J. Physiol.-Gastrointest. Liver Physiol..

[82] Garro A.J., McBeth D.L., Lima V., Lieber C.S. (1991). Ethanol Consumption Inhibits Fetal DNA Methylation in Mice: Implications for the Fetal Alcohol Syndrome. Alcohol Clin. Exp. Res..

[83] Bonsch D., Lenz B., Fiszer R., Frieling H., Kornhuber J., Bleich S. (2006). Lowered DNA Methyltransferase (DNMT-3b) MRNA Expression Is Associated with Genomic DNA Hypermethylation in Patients with Chronic Alcoholism. J. Neural Transm..

[84] Streissguth A.P., Aase J.M., Clarren S.K., Randels S.P., LaDue R.A., Smith D.F. (1991). Fetal Alcohol Syndrome in Adolescents and Adults. JAMA-J. Am. Med. Assoc..

[85] Halsted C.H., Medici V. (2012). Aberrant Hepatic Methionine Metabolism and Gene Methylation in the Pathogenesis and Treatment of Alcoholic Steatohepatitis. Int. J. Hepatol..

[86] Nagre N.N., Subbanna S., Shivakumar M., Psychoyos D., Basavarajappa B.S. (2015). CB1-Receptor Knockout Neonatal Mice Are Protected Against Ethanol-Induced Impairments of DNMT1, DNMT3A and DNA Methylation. J. Neurochem..

[87] Otero N.K.H., Thomas J.D., Saski C.A., Xia X., Kelly S.J. (2012). Choline supplementation and dna methylation in the hippocampus and prefrontal cortex of rats exposed to alcohol during development. Alcohol Clin. Exp. Res..

[88] Perkins A., Lehmann C., Lawrence C.R., Kelly S.J. (2013). Alcohol Exposure during Development: Impact on the Epigenome. Int. J. Dev. Neurosci..

[89] Liu Y., Balaraman Y., Wang G., Nephew K.P., Zhou F.C. (2009). Alcohol Exposure Alters DNA Methylation Profiles in Mouse Embryos at Early Neurulation. Epigenetics.

[90] Raffin-Sanson M.L., de Keyzer Y., Bertagna X. (2003). Proopiomelanocortin, a Polypeptide Precursor with Multiple Functions: From Physiology to Pathological Conditions. Eur. J. Endocrinol..

[91] Bekdash R.A., Zhang C., Sarkar D.K. (2013). Gestational Choline Supplementation Normalized Fetal Alcohol-Induced Alterations in Histone Modifications, DNA Methylation, and Proopiomelanocortin (POMC) Gene Expression in β-Endorphin-Producing POMC Neurons of the Hypothalamus. Alcohol. Clin. Exp. Res..

[92] Gangisetty O., Sinha R., Sarkar D.K. (2019). Hypermethylation of Proopiomelanocortin and Period 2 Genes in Blood Are Associated with Greater Subjective and Behavioral Motivation for Alcohol in Humans. Alcohol Clin. Exp. Res..

[93] Govorko D., Bekdash R.A., Zhang C., Sarkar D.K. (2012). Male Germline Transmits Fetal Alcohol Adverse Effect on Hypothalamic Proopiomelanocortin Gene across Generations Dmitry. Biol. Psychiatry.

[94] Sarkar D.K., Gangisetty O., Wozniak J.R., Eckerle J.K., Georgieff M.K., Foroud T.M., Wetherill L., Wertelecki W., Chambers C.D., Riley E. (2019). Persistent Changes in Stress-Regulatory Genes in Pregnant Women or Children Exposed Prenatally to Alcohol. Alcohol. Clin. Exp. Res..

[95] Gangisetty O., Chaudhary S., Palagani A., Sarkar D.K. (2022). Transgenerational Inheritance of Fetal Alcohol Effects on Proopiomelanocortin Gene Expression and Methylation, Cortisol Response to Stress, and Anxiety-like Behaviors in Offspring for Three Generations in Rats: Evidence for Male Germline Transmission. PLoS ONE.

[96] American Psychiatric Association (2013). The Diagnostic and Statistical Manual of Mental Disorders: DSM-5TM.

[97] The National Child Traumatic Stress Network. https://www.nctsn.org.

[98] Heim C., Newport D.J., Mletzko T., Miller A.H., Nemeroff C.B. (2008). The Link between Childhood Trauma and Depression: Insights from HPA Axis Studies in Humans. Psychoneuroendocrinology.

[99] Saunders B.E., Adams Z.W. (2014). Epidemiology of Traumatic Experiences in Childhood. Child Adolesc. Psychiatr. Clin. N. Am..

[100] McGowan P.O., Sasaki A., D’Alessio A.C., Dymov S., Labonté B., Szyf M., Turecki G., Meaney M.J. (2009). Epigenetic Regulation of the Glucocorticoid Receptor in Human Brain Associates with Childhood Abuse. Nat. Neurosci..

[101] Painter R.C., Roseboom T.J., Bleker O.P. (2005). Prenatal Exposure to the Dutch Famine and Disease in Later Life: An Overview. Reprod. Toxicol..

[102] Yehuda R., Daskalakis N.P., Bierer L.M., Bader H.N., Klengel T., Holsboer F., Binder E.B. (2016). Holocaust Exposure Induced Intergenerational Effects on FKBP5 Methylation. Biol. Psychiatry.

[103] Hompes T., Izzi B., Gellens E., Morreels M., Fieuws S., Pexsters A., Schops G., Dom M., Van Bree R., Freson K. (2014). Investigating the Influence of Maternal Cortisol and Emotional State during Pregnancy on the DNA Methylation Status of the Glucocorticoid Receptor Gene (NR3C1) Promoter Region in Cord Blood. J. Psychiatr. Res..

[104] Mulligan C.J., D’Errico N., Stees J., Hughes D.A. (2012). Methylation Changes AtNR3C1in Newborns Associate with Maternal Prenatal Stress Exposure and Newborn Birth Weight. Epigenetics.

[105] Oberlander T.F., Weinberg J., Papsdorf M., Grunau R., Misri S., Devlin A.M. (2008). Prenatal Exposure to Maternal Depression, Neonatal Methylation of Human Glucocorticoid Receptor Gene (NR3C1) and Infant Cortisol Stress Responses. Epigenetics.

[106] Radtke K.M., Ruf M., Gunter H.M., Dohrmann K., Schauer M., Meyer A., Elbert T. (2011). Transgenerational Impact of Intimate Partner Violence on Methylation in the Promoter of the Glucocorticoid Receptor. Transl. Psychiatry.

[107] Yang H.H., Hu N., Wang C., Ding T., Dunn B.K., Goldstein A.M., Taylor P.R., Lee M.P. (2010). Influence of Genetic Background and Tissue Types on Global DNA Methylation Patterns. PLoS ONE.

[108] Waterland R.A., Kellermayer R., Laritsky E., Rayco-Solon P., Harris R.A., Travisano M., Zhang W., Torskaya M.S., Zhang J., Shen L. (2010). Season of Conception in Rural Gambia Affects DNA Methylation at Putative Human Metastable Epialleles. PLoS Genet..

[109] Cao-Lei L., Massart R., Suderman M.J., Machnes Z., Elgbeili G., Laplante D.P., Szyf M., King S. (2014). DNA Methylation Signatures Triggered by Prenatal Maternal Stress Exposure to a Natural Disaster: Project Ice Storm. PLoS ONE.

[110] Yehuda R., Bierer L.M., Schmeidler J., Aferiat D.H., Breslau I., Dolan S. (2000). Low Cortisol and Risk for PTSD in Adult Offspring of Holocaust Survivors. Am. J. Psychiatry.

[111] Bierer L.M., Bader H.N., Daskalakis N.P., Lehrner A., Provençal N., Wiechmann T., Klengel T., Makotkine I., Binder E.B., Yehuda R. (2020). Intergenerational Effects of Maternal Holocaust Exposure on FKBP5 Methylation. Am. J. Psychiatry.

[112] Yehuda R., Daskalakis N.P., Lehrner A., Desarnaud F., Bader H.N., Makotkine I., Flory J.D., Bierer L.M., Meaney M.J. (2014). Influences of Maternal and Paternal PTSD on Epigenetic Regulation of the Glucocorticoid Receptor Gene in Holocaust Survivor Offspring. Am. J. Psychiatry.

[113] Burger G.C.E., Drummond J.C., Sandstead H.R., Staatsuitgeverij’s G. (1950). Malnutrition and Starvation in Western Netherlands September 1944–July 1945. Part I. Part II: Appendices. J. Am. Med. Assoc..

[114] De Rooij S.R., Bleker L.S., Painter R.C., Ravelli A.C., Roseboom T.J. (2021). Lessons Learned from 25 Years of Research into Long Term Consequences of Prenatal Exposure to the Dutch Famine 1944–45: The Dutch Famine Birth Cohort. Int. J. Environ. Health Res..

[115] Bleker L.S., De Rooij S.R., Painter R.C., Ravelli A.C., Roseboom T.J. (2021). Cohort Profile: The Dutch Famine Birth Cohort (DFBC)—A Prospective Birth Cohort Study in the Netherlands. BMJ Open.

[116] Heijmans B.T., Tobi E.W., Stein A.D., Putter H., Blauw G.J., Susser E.S., Slagboom P.E., Lumey L.H. (2008). Persistent Epigenetic Differences Associated with Prenatal Exposure to Famine in Humans. Proc. Natl. Acad. Sci. USA.

[117] Tobi E.W., Lumey L.H., Talens R.P., Kremer D., Putter H., Stein A.D., Slagboom P.E., Heijmans B.T. (2009). DNA Methylation Differences after Exposure to Prenatal Famine Are Common and Timing- and Sex-Specific. Hum. Mol. Genet..

[118] Veenendaal M.V., Costello P.M., Lillycrop K.A., de Rooij S.R., van der Post J.A., Bossuyt P.M., Hanson M.A., Painter R.C., Roseboom T.J. (2012). Prenatal Famine Exposure, Health in Later Life and Promoter Methylation of Four Candidate Genes. J. Dev. Orig. Health Dis..

[119] Harris A., Seckl J. (2011). Glucocorticoids, Prenatal Stress and the Programming of Disease. Horm. Behav..

[120] Coelho R., Viola T.W., Walss-Bass C., Brietzke E., Grassi-Oliveira R. (2014). Childhood Maltreatment and Inflammatory Markers: A Systematic Review. Acta Psychiatr. Scand..

[121] Juengst E.T., Fishman J.R., McGowan M.L., Settersten R.A. (2014). Serving Epigenetics before Its Time. Trends Genet..

[122] Yehuda R., Lehrner A., Bierer L.M. (2018). The Public Reception of Putative Epigenetic Mechanisms in the Transgenerational Effects of Trauma. Environ. Epigenet..

[123] Carey B. Can We Really Inherit Trauma?. https://www.nytimes.com/2018/12/10/health/mind-epigenetics-genes.html.

[124] Fitzgerald M., London-Johnson A., Gallus K.L. (2020). Intergenerational Transmission of Trauma and Family Systems Theory: An Empirical Investigation. J. Fam. Ther..

[125] Harding K.D., Turner K., Howe S.J., Bagshawe M.J., Flannigan K., Mela M., McMorris C.A., Badry D. (2022). Caregivers’ Experiences and Perceptions of Suicidality among Their Children and Youth with Fetal Alcohol Spectrum Disorder. Front. Psychiatry.

[126] Dickson J., Stewart M. (2021). Risk, Rights and Deservedness: Navigating the Tensions of Gladue, Fetal Alcohol Spectrum Disorder and Settler Colonialism in Canadian Courts. Behav. Sci. Law.

[127] King B., Fallon B., Boyd R., Black T., Antwi-Boasiako K., O’Conner C. (2017). Factors Associated with Racial Differences in Child Welfare Investigative Decision-Making in Ontario, Canada. Child Abuse Negl..

[128] Pei J., Carlson E., Tremblay M., Poth C. (2019). Exploring the Contributions and Suitability of Relational and Community-Centered Fetal Alcohol Spectrum Disorder (FASD) Prevention Work in First Nation Communities. Birth Defects Res..

[129] Felitti V.J., Anda R.F., Nordenberg D., Williamson D.F., Spitz A.M., Edwards V., Koss M.P., Marks J.S. (1998). Relationship of Childhood Abuse and Household Dysfunction to Many of the Leading Causes of Death in Adults. Am. J. Prev. Med..

[130] Andersson S.-O., Annerback E.-M., Sondergaard H.P., Hallqvist J., Kristiansson P. (2021). Adverse Childhood Experiences Are Associated with Choice of Partner, Both Partners’ Relationship and Psychosocial Health as Reported One Year after Birth of a Common Child. A Cross-Sectional Study. PLoS ONE.

[131] Kambeitz C., Klug M.G., Greenmyer J., Popova S., Burd L. (2019). Association of Adverse Childhood Experiences and Neurodevelopmental Disorders in People with Fetal Alcohol Spectrum Disorders (FASD) and Non-FASD Controls. BMC Pediatr..

[132] Griffin G. (2020). Defining Trauma and a Trauma-Informed COVID-19 Response. Psychol. Trauma Theory Res. Pract. Policy.

[133] Government of Canada (2015). Canada’s Residential Schools: The Final Report of the Truth and Reconciliation Commission of Canada.

[134] Buller M., Audette M., Robinson Q., Evolfson B. (2019). Reclaiming Power and Place. The Final Report of the National Inquiry into Missing and Murdered Indigenous Women and Girls Reclaiming Power and Place Volume 1a.

[135] Burczycka M. Section 1: Profile of Canadian Adults Who Experienced Childhood Maltreatment. https://www150.statcan.gc.ca/n1/pub/85-002-x/2017001/article/14698/01-eng.htm.

[136] Greenwood M., de Leeuw S., Lindsay N.M. (2015). Determinants of Indigenous Peoples’ Health: Beyond the Social.

[137] Ryan J., Chaudieu I., Ancelin M.L., Saffery R. (2016). Biological Underpinnings of Trauma and Post-Traumatic Stress Disorder: Focusing on Genetics and Epigenetics. Epigenomics.

[138] Youssef N.A., Lockwood L., Su S., Hao G., Rutten B.P.F. (2018). The Effects of Trauma, with or without PTSD, on the Transgenerational DNA Methylation Alterations in Human Offsprings. Brain Sci..

[139] Matthews S.G., Phillips D.I. (2012). Transgenerational Inheritance of Stress Pathology. Exp. Neurol..

[140] Maylea C. (2020). The End of Social Work. Br. J. Soc. Work.

[141] Combs-Orme T. (2013). Epigenetics and the Social Work Imperative. Soc. Work.

[142] White S.J., Wastell D.G. (2016). Epigenetics Prematurely Born(e): Social Work and the Malleable Gene. Br. J. Soc. Work.

[143] Wastell D., White S. (2017). Blinded by Science. The Social Implications of Epigenetics and Neuroscience.

[144] Lappé M. (2016). Epigenetics, Media Coverage, and Parent Responsibilities in the Post-Genomic Era. Curr. Genet. Med. Rep..

[145] Liberman N., Wang S.Y., Greer E.L. (2019). Transgenerational Epigenetic Inheritance: From Phenomena to Molecular Mechanisms. Curr. Opin. Neurobiol..

[146] Gladkova M.G., Leidmaa E., Anderzhanova E.A. (2023). Epidrugs in the Therapy of Central Nervous System Disorders: A Way to Drive On?. Cells.

[147] Motz M., Andrews N.C.Z., Bondi B.C., Leslie M., Pepler D.J. (2019). Addressing the Impact of Interpersonal Violence in Women Who Struggle with Substance Use through Developmental-Relational Strategies in a Community Program. Int. J. Environ. Res. Public Health.

